# Association between self-reported oral health and cognitive function among the community-dwelling elderly in Jinan, China: the mediating effect of life satisfaction

**DOI:** 10.3389/fpsyg.2023.1116325

**Published:** 2023-05-25

**Authors:** Guangwen Liu, Zhongqian Lu, Ying Shan, Jieru Wang, Xinfei Shi, Di Zong, Shixue Li, Fanlei Kong

**Affiliations:** ^1^Centre for Health Management and Policy Research, School of Public Health, Cheeloo College of Medicine, Shandong University, Jinan, China; ^2^NHC Key Lab of Health Economics and Policy Research, Shandong University, Jinan, China

**Keywords:** life satisfaction, cognitive function, community-dwelling elderly, mediating effect, self-reported oral health

## Abstract

**Background:**

Deterioration of self-reported oral health and decline in cognitive function are two main adverse health outcomes experienced by the older adults. Little evidence was found on the psychosocial mechanism between self-reported oral health and cognitive function. This study explores the association between self-reported oral health and cognitive function and examines the mediating effect of life satisfaction among the community-dwelling elderly in Jinan, China.

**Methods:**

A total of 512 older individuals (60+) were included in the study. Cognitive function was assessed using the Chinese version of the Mini-Mental State Examination scale (MMSE), and self-reported oral health was measured using the Chinese version of the Geriatric Oral Health Assessment Index (GOHAI). Pearson correlation analysis was used to determine the relationship between self-reported oral health, life satisfaction, and cognitive function. Multivariate linear regression analysis was conducted to explore the possible effect of covariates. Structural equation modelling and Bootstrap analyses were conducted to verify the mediating role of life satisfaction.

**Results:**

The mean MMSE score was 25.65 ± 4.42. Better self-reported oral health was significantly associated with a higher level of life satisfaction, and those with higher life satisfaction experienced better cognitive function. Age, educational level and source of living expenses were found to be cofounding variables. Life satisfaction partially mediated the effect of self-reported oral health on cognitive function (95% confidence interval [CI]: 0.010 to 0.075). The mediating effect of life satisfaction accounted for 24% of the total effect.

**Conclusion:**

The level of cognitive function was relatively high. Self-reported oral health was positively associated with cognitive function, and the mediating effect of life satisfaction was proven to exist in the community-dwelling older individuals. Early screening for oral diseases and a greater focus on life satisfaction are recommended.

## 1. Introduction

As life expectancy has increased globally, population aging has inevitably followed. Data from *World Health Statistics 2021* showed that the worldwide average life expectancy at birth has increased by 6.5 years in the past two decades, reaching 73.3 years in 2019 (World Health Organization, 2021). The latest world demographic data showed that people aged 65 years and older now accounted for 10% of the global population (United Nations, 2022). Huge progress in the area of medical technology has meant that the phenomenon of enhanced life expectancy and population aging has also extended to China. Data from China’s Seventh National Census showed that older Chinese adults aged 60 years and above accounted for 18.7% of China’s population in November 2020 (National Bureau of Statistics, 2021). As individuals step into their later life, a wide range of physiological problems arise, such as multimorbidity, limited activities in daily living, sleep disorders, and cognitive decline (Eshkoor et al., 2015). Identifying risk factors for the cognitive impairment and developing target interventions to improve the physical function of older people deserves greater attention from healthcare professionals.

Cognitive impairment, a chronic disease characterized by decline in memory, attention, learning and other cognitive functions, created a substantial disease burden for older adults. According to the Global Burden of Disease (GBD) 2019, Alzheimer’s disease and other dementias were the fourth leading cause of disability-adjusted life years (DALYs) among individuals aged 75 years and older, contributing 18.4 million DALYs of the total (GBD, 2019 Diseases and Injuries Collaborators, 2020). A national cross-sectional study in China reported that the incidence of cognitive impairment was 15.5% among people over the age of 60 (Jia et al., 2020). Previous studies have revealed various factors that influence cognitive function in older people. Aside from increased age, other sociodemographic variables (such as gender, marital status, educational level, occupation, and so on) (Fei et al., 2009), physical problems (Wang et al., 2020), mental disorders (Shimada et al., 2014; Boss et al., 2015), living habits (Kirk-Sanchez and McGough, 2014; Zhang Q. et al., 2019), and social resources factors (Zunzunegui et al., 2003) were all found to have an influence on geriatric cognitive function.

Oral health was another health outcome that deteriorated with age (Raphael, 2017). Oral health among older adults could be measured using clinical questions concerning caries, missing teeth, dentures, bleeding gums, and periodontal disease, as well as multiple-item scales such as the Geriatric Oral Health Assessment Index (GOHAI) (Atchison and Dolan, 1990) and the Oral Health Impact Profile (OHIP-14) (Slade, 1997). Many researchers have explored the association between oral health and cognitive function. Stewart et al. found that older adults who developed more severe gingival inflammation were 1.55 times more likely to report cognitive impairment (Stewart et al., 2013). A Japanese study suggested that community-dwelling elderly with fewer teeth were more likely to develop all-cause dementia (Takeuchi et al., 2017). Research on older Korean individuals also indicated that poor self-reported oral health (measured by higher scores in GOHAI) was related to cognitive impairment (Lee et al., 2020). A cross-sectional study of older Chinese in Shanghai revealed that participants who had lost more than 16 teeth were 1.56 times more likely to have dementia compared to those who lost 0–3 teeth (Luo et al., 2015). A study conducted in Taiwan also found that moderate and severe periodontitis was associated with poor cognitive performance (Sung et al., 2019). However, to date, no study has used multiple-item scales to examine the association between self-reported oral health and cognitive function among the older Chinese people.

Life satisfaction was defined as “an individual’s subjective feeling and evaluation based on his/her own living conditions” (Larson, 1979). Better life satisfaction was found to be related to several beneficial health outcomes concerning physical function, psychosocial wellbeing and health behaviors in previous studies (Kim et al., 2021; Luo et al., 2022). A population-based study revealed that older adults with dementia or cognitive impairment no dementia had lower life satisfaction scores than those who were cognitively intact (St John and Montgomery, 2010). A 17-year longitudinal study of cognitive aging found that an increase in life satisfaction was associated with better cognitive performance among older adults without depression (Rabbitt et al., 2008). Higher levels of subjective life satisfaction were also found to exert a significant mediating effect in delaying cognitive decline and dementia onset in Asian countries (Lee, 2018).

The association between self-reported oral health and life satisfaction or other psychosocial variables has been widely established. Locker et al. conducted a seven-year cohort study and revealed the existence of a longitudinal relationship between self-perceived oral health and life satisfaction (Locker et al., 2000). Another study also demonstrated that self-rated oral health could predict life satisfaction and self-esteem (Benyamini et al., 2004). Difficulty in chewing function and dry mouth were also found to be associated with the lower frequency of going out for social interaction (Mikami et al., 2019). However, few studies have focused on the mediating effect of psychosocial factors on the relationship between self-reported oral health and cognitive function. Existing studies on the underlying mechanism between oral health and cognitive function have mainly focused on physiological aspects, such as oral microbiome dysbiosis (Orr et al., 2020), inflammation response (Gorelick, 2010), and nutrition imbalance (Kossioni, 2018). Therefore, the mediating role of psychosocial variables on the link between self-reported oral health and cognitive function deserved more attention in future studies.

To conclude, to date, no study has explored the relationship between self-reported oral health, life satisfaction and cognitive function simultaneously among the community-dwelling elderly in China. Therefore, this study aims to examine the relationship between self-reported oral health and cognitive function, and to explore the mediating effect of life satisfaction on this association among the community-dwelling elderly in Jinan, China.

## 2. Materials and methods

### 2.1. Data collection and the research subjects

The data were collected in August 2020 in Jinan, Shandong Province, China. Shandong Province lies in the east of China, which has the largest number of older population in China (Shandong Provincial Government, 2021). Jinan is the capital of Shandong Province, with a gross domestic product of CNY 1.01 trillion (approximately USD 158 billion) in 2020 (Jinan Municipal Bureau of Statistics, 2021). Moreover, the number of older population in Jinan ranks fourth among Shandong Province (Shandong Provincial Bureau of Statistics, 2021). Taking the target population is the older adults in to consideration, it was more feasible and reasonable to choose Jinan City as the sample place. As of 1 July 2020, Jinan has jurisdiction over 10 districts and two counties (132 sub-districts and 29 towns) (Jinan Municipal Government, 2020). By the end of 2019, the local resident population was 8.91 million—an increase of 0.78% over the previous year—while the registered population was 7.98 million, an increase of 1.46% (Jinan Municipal Government, 2021). The total urban population in Jinan City in 2019 was 5.96 million (Ministry of Housing and Urban-Rural Development of China, 2020), which included 4.35 million urban residents and 1.6 million urban temporary residents; this study included Jinan City urban elderly who were older than 60 years of age.

The method of multi-stage cluster random sampling was used to recruit participants. In the first stage, two districts were chosen among 10 districts as the primary sampling units, comprehensively considering economic development and geographic location. In the second stage, two sub-districts were selected from each primary sampling unit as the secondary sampling units, which means that one sub-district was chosen from each of the previously selected districts. In the third stage, two communities were selected from the secondary sampling units, which means that one community was chosen from each of the previously selected sub-districts. All the urban elderly in these two communities constituted the total sample of this study.

The inclusion criteria of the respondents were adults aged 60 years or above living in Jinan City who were informed about the content of this study and could communicate. To obtain complete and accurate data, this study excluded participants who were unwilling to cooperate with the interview owing to their physical condition or other reasons.

According to a previous study (Shao et al., 2018), the following formula about the sample size calculation was used in this study.


$$n=\mathrm{deff}\frac{u_{\alpha }^{2}\;p\left(1-p\right)}{\varepsilon^{2}}$$

In the above formula, the design efficiency deff=2.5, the $u_{\alpha }^{2}$ represented the value of u statistics in the confidence level of 1-α. When α = 0.05, the value of u statistics was 1.96; the margin of error ε = 6%, the prevalence of cognitive impairment among the older adults aged 60 years or older in China was *p* = 21.5% (Jia et al., 2020), and the non-response rate was 10%. Thus, a minimum of 496 samples would be needed for the research.

Thirty-two university students became the investigators after receiving training concerning the study background, questionnaire contents, and social survey techniques. Twenty-minute face-to-face interviews were conducted between the investigators and the participants to collect the data. A total of 522 elderly people were initially chosen and interviewed; however, 10 were excluded from the sample owing to obvious logical errors or incomplete questionnaires. The data from 512 elderly individuals were thus analyzed.

### 2.2. Measurements

#### 2.2.1. Dependent variable

Cognitive function was evaluated using the Chinese version of the Mini-Mental State Examination scale (MMSE). The MMSE was first invented by Folstein (Folstein et al., 1975) and then translated into Chinese by Katzman (Katzman et al., 1988). Respondents were asked to answer 30 questions pertaining to the following five dimensions: orientation, registration, attention and calculation, recall, and language. They would get one point if answering these questions correctly. Thus, the total score for the test ranges from 0 to 30 with higher scores indicating better cognitive function. This scale has been widely used in previous studies in China, showing good reliability and validity (Lv et al., 2019; Chen et al., 2021).

#### 2.2.2. Independent variables

Self-reported oral health.

Self-reported oral health was evaluated using the Chinese version of the Geriatric Oral Health Assessment Index (GOHAI). The GOHAI was first invented by Atchison to measure the effects of oral diseases or oral health problems in older adults (Atchison and Dolan, 1990). In China, the GOHAI was first translated into Cantonese (a dialect prevailing in Hong Kong and Guangdong Province) by Hong Kong based scholars (Wong et al., 2002). For the convenience of most regions of mainland China, Wang et al. later translated the English version of the GOHAI into Mandarin Chinese and verified its reliability and validity (Wang and Ling, 2011). This study adopted the Chinese version of the GOHAI translated by Wang et al. It contains 12 items divided into three dimensions: physical function, psychosocial function, and pain or discomfort. Respondents were asked to select one choice on a five-point Likert scale ranging from 1 (“Always”) to 5 (“Never”). Thus, the Chinese version of the GOHAI has a total score ranging from 12 to 60, with higher scores indicating better self-reported oral health. Participants were further divided into three categories, as described in a previous study (Yu et al., 2008). Participants who received a total score of 12–50 were deemed to have a low level of self-reported oral health. Those who obtained a score of 51–56 were deemed as having a moderate level of self-reported oral health, while those who had a summary score of 57 or above were recognized as a high level of self-reported oral health. The Cronbach’s α and Kaiser-Meyer-Olkin (KMO) value of this scale were 0.854 and 0.86 respectively, indicating an acceptable internal consistency reliability and structural validity.

Life Satisfaction.

Life satisfaction was assessed by asking individuals the following questions: “Were you satisfied with getting help from others?,” “Were you satisfied with interpersonal relationships?” and “Were you satisfied with your marriage?” Responses to these questions were rated on a five-point Likert scale ranging from 1 (“Very unsatisfied”) to 5 (“Very satisfied”). However, as few respondents selected “Very unsatisfied” or “Quite unsatisfied” (the proportion were 2.5, 1.0, and 1.4% for each question), the answer was recoded into 1 (“General or below”), 2 (“Quite satisfied”) and 3 (“Very satisfied”). The Cronbach’s α and Kaiser-Meyer-Olkin (KMO) value of the above variables were 0.841 and 0.643 respectively, indicating an acceptable internal consistency reliability and structural validity.

#### 2.2.3. Covariables

Respondents’ gender, age, marital status, educational level, monthly income, employment status, source of living expenses, physical health, mental health, outpatient service last year, and inpatient service last year were introduced in the current study. Gender was divided into the categories of male or female; age was divided into groupings of 60–69 years, 70–79 years, or 80 years or above; marital status was divided into currently married or single (including widowed, divorced, unmarried and other conditions); educational level was divided into illiterate, primary school graduate, middle school graduate, and university or above graduate; monthly income was divided into four groups according to the quartile; employment status was coded into currently employed, retired and unemployed; source of living expenses was coded into from their own or spouses, from their children or other relatives and from basic living allowances. Furthermore, the Short-Form Health Survey (SF-12) was also used to measure their physical and mental health (Gandek et al., 1998). SF-12 scores were divided into physical component summary (PCS) and mental component summary (MCS) scores (Ware et al., 1995) and both of them could be dichotomized by the cut-off point of the first quartile (Khedmat et al., 2007). Following a previous study did, participants whose PCS/MCS scores were lower than the first quartile were defined as poor physical/mental health while those whose scores were higher than the first quartile were defined as good physical/mental health (Sampogna et al., 2019). Finally, outpatient service as well as inpatient service last was categorized into yes or no.

### 2.3. Statistical analysis

Data were presented as the mean and standard deviation for continuous variables, and as frequency and percentage for categorical variables. T-test and one-way Analysis of Variation (ANOVA) were used to compare the difference in MMSE scores among the different subgroups of sociodemographic variables, self-reported oral health, and life satisfaction indicators. Furthermore, Pearson and spearman correlation analyses were conducted to examine the association between the GOHAI, life satisfaction indicators and MMSE scores. According to previous studies, the correlation was considered as negligible when the coefficient was below 0.10 and deemed to be weak when between 0.10 and 0.39 (Schober et al., 2018). Multivariate linear regression analysis was conducted to determine the potential impact of confounding variables on the cognitive function. Statistical significance was set at *p* ≤ 0.05. All analyses were conducted using SPSS 24.0 (IBM Corp, Armonk, NY, United States).

The mediating effect was tested using structural equation modelling (SEM) analysis. Two hypothesized models were established. The first model contained the independent variable and dependent variable. In the second model, the possible mediator was further introduced. Standardized effects over the arrows were obtained using maximum likelihood estimation. The mediating effect was examined by the bootstrap method with 2,000 samples (Fritz and MacKinnon, 2007). The mediating effect was considered to be established if the 95% confidence interval (CI) of the indirect effect excluded zero. In addition, several model fitness indices were used to decide whether the hypothesized models fitted the empirical data (Bollen, 1989; Hu and Bentler, 1999): *χ^2^*/*df*, comparative fit index (CFI), goodness fit index (GFI), adjusted goodness fit index (AGFI) and root mean square error of approximation (RMSEA). The current model was well fitted when *χ*^2^/*df* < 5, CFI > 0.90, GFI > 0.90, AGFI >0.90 and RMSEA <0.08. Statistical significance was set at *p* ≤ 0.05. All SEM analyses were conducted using AMOS 24.0 (IBM Corp, Armonk, NY, United States).

### 2.4. Ethical considerations

Medical ethics approval of this study was approved by the Ethical Committee of School of Public Health, Shandong University (No. 20180225). Informed consent for the data collection and the use of the data was obtained from all subjects.

## 3. Results

### 3.1. Fundamental characteristics of the study participants

Basic features and self-reported oral health status, life satisfaction, and cognitive function among the participants were demonstrated in Table 1. The average score of MMSE was 25.65 ± 4.62. Respondents were predominantly female (64.5%), 60 to 69 years old (52.1%), currently married (76.2%), at a middle school educational level (61.9%), retired (75.3%), received living expenses from their own or spouses (86.1%), and reported good physical health (75.0%) and mental health (73.8%). Concerning the access to care, over 70% of the individuals did not receive outpatient service last year and around three fourth did not get inpatient service last year. Regarding self-reported oral health, 57.0% of respondents had a high GOHAI score, indicating a high level of self-reported oral health, while over one-fifth had a low level of self-reported oral health. As for life satisfaction, most participants were very satisfied with getting help, interpersonal relationship, and their marriage (the proportions were 46.5, 49.4, and 52.3% respectively). The results of the t-test or one-way ANOVA in Table 1 showed that there were statistical differences in the participants’ MMSE scores with different subgroups of age, marital status, educational level, monthly income, employment status, source of living expenses, physical health, inpatient service, self-reported oral health, satisfaction with getting help, satisfaction with interpersonal relationship, and satisfaction with marriage. Specifically, those who aged 60–69 years, were currently married, were university or above graduate, had the highest monthly income, retired from work, made their livings by themselves or spouses, reported good physical health, and did not receive inpatient service last year scored better on MMSE. Older adults who had high level of self-reported oral health and were very satisfied with getting help, interpersonal relationship and marriage reported better cognitive function.

**Table 1:** Basic characteristics and univariate analysis on the association between self-reported oral health, life satisfaction, and cognitive function among the community-dwelling elderly in Jinan, China.

Variables	*N* (%)	Mean score of MMSE (SD)	*t*/*F*	*p*-value
Total	512 (100)	25.65 (4.62)		
Gender			0.178^c^	0.859
Male	182 (35.5)	25.70 (4.44)		
Female	330 (64.5)	25.63 (4.72)		
Age			34.611^d^	**<0.001**
60–69	267 (52.1)	26.88 (3.56)		
70–79	159 (31.1)	25.33 (4.53)		
80 or above	86 (16.8)	22.44 (5.93)		
Marital status			5.006^c^	**<0.001**
Currently married	390 (76.2)	26.21 (3.99)		
Single^a^	122 (23.8)	23.87 (5.89)		
Educational level			36.431^d^	**<0.001**
Illiterate	44 (8.6)	20.43 (6.37)		
Primary school	101 (19.7)	24.07 (4.69)		
Middle school	317 (61.9)	26.56 (3.81)		
University or above	50 (9.8)	27.72 (3.04)		
Monthly income^b^			5.242^d^	**0.001**
Q1	154 (30.1)	24.70 (5.16)		
Q2	105 (20.5)	26.48 (3.84)		
Q3	147 (28.7)	25.35 (4.73)		
Q4	106 (20.7)	26.64 (4.02)		
Employment status			12.065^d^	**<0.001**
Currently employed	24 (4.7)	26.08 (4.20)		
Retired	386 (75.3)	26.15 (4.29)		
Unemployed	102 (20.0)	23.69 (5.38)		
Source of living expenses			17.114^d^	**<0.001**
From their own or spouses	441 (86.1)	26.10 (4.11)		
From their children or other relatives	63 (12.3)	22.57 (6.55)		
From basic living allowances	8 (1.6)	25.38 (4.10)		
Physical health (PCS)			−2.011^c^	**0.045**
Poor	128 (25.0)	24.95 (5.16)		
Good	384 (75.0)	25.89 (4.40)		
Mental health (MCS)			−1.060^c^	0.290
Poor	134 (26.2)	25.29 (5.35)		
Good	378 (73.8)	25.78 (4.33)		
Outpatient service last year			−0.476^c^	0.634
No	366 (71.5)	25.59 (4.80)		
Yes	146 (28.5)	25.81 (4.16)		
Inpatient service last year			2.087^c^	**0.037**
No	383 (74.8)	25.90 (4.44)		
Yes	129 (25.2)	24.92 (5.06)		
Self-reported oral health			4.946^d^	**0.007**
Low	115 (22.5)	24.48 (5.53)		
Moderate	105 (20.5)	25.86 (3.81)		
High	292 (57.0)	26.04 (4.43)		
Satisfaction with getting help			7.612^d^	**0.001**
General or below	55 (10.7)	23.71 (6.30)		
Quite satisfied	219 (42.8)	25.44 (4.41)		
Very satisfied	238 (46.5)	26.30 (4.22)		
Satisfaction with interpersonal relationship			5.801 ^d^	**0.003**
General or below	46 (9.0)	23.59 (6.94)		
Quite satisfied	213 (41.6)	25.60 (4.23)		
Very satisfied	253 (49.4)	26.08 (4.32)		
Satisfaction with marriage			3.782^d^	**0.023**
General or below	51 (10.0)	24.22 (6.20)		
Quite satisfied	193 (37.7)	25.46 (4.37)		
Very satisfied	268 (52.3)	26.07 (4.39)		

MMSE, Mini-Mental State Examination; SD, Standard deviation; PCS, Physical component summary; MCS, Mental component summary.

a Single included those who were unmarried (10, 1.5%), divorced (5, 0.8%), and widowed (58, 8.8%).

b Q1 was the poorest and Q4 was the richest.

c *t*-value.

d *F*-value. Bold values indicated statistical significance (*p* ≤ 0.05).

### 3.2. Correlation analysis on the key variables

Table 2 presented the correlation matrix between self-reported oral health, the three indicators of life satisfaction, and cognitive function. Cognitive function was found to be positively and weakly related to self-reported oral health (r = 0.121, *p* ≤ 0.01). In addition, significant and positive association was found between cognitive function and satisfaction with getting help (*r* = 0.164, *p* ≤ 0.001), satisfaction with interpersonal relationship (*r* = 0.116, *p* ≤ 0.01) as well as satisfaction with marriage (*r* = 0.116, *p* ≤ 0.01), although the associations between these variables were weak. It was also found that self-reported oral health was positively and weakly related to satisfaction with getting help (*r* = 0.194, *p* ≤ 0.001), interpersonal relationship (*r* = 0.193, *p* ≤ 0.001). Moreover, the relationship between self-reported oral health and marital satisfaction was significant, but the correlation was negligible (*r* = 0.088, *p* ≤ 0.05).

**Table 2:** Correlation between the indicators of independent variables and cognitive function among the community-dwelling elderly in Jinan, China.

Variables	1	2	3	4	5
1. Self-reported oral health	1				
2. Satisfaction with getting help	0.194^a^^***^	1			
3. Satisfaction with interpersonal relationship	0.193^a^^***^	0.873^a^^***^	1		
4.Satisfaction with marriage	0.088^a^^*^	0.567^a^^***^	0.535^a^^***^	1	
5. Cognitive function	0.121^b^^**^	0.164^a^^***^	0.116^a^^**^	0.116^a^^**^	1

SD: Standard deviation; *p ≤ 0.05, **p ≤ 0.01, ***p ≤ 0.001.

a Pearson correlation coefficient.

b Spearman correlation coefficient.

### 3.3. Multivariate linear regression analysis

Table 3 demonstrated the association of cognitive function, key variables and confounding factors. Self-reported oral health was significantly related to cognitive function when the covariates were included in Model 2 (β = 0.101, *p* = 0.040). However, when three indicators of life satisfaction were introduced in Model 3 to 5 respectively, the effect size of self-reported oral health on cognitive function became small and insignificant (β = 0.082, *p* = 0.096 in Model 3; β = 0.086, *p* = 0.083 in Model 4; β = 0.094, *p* = 0.054 in Model 5). Furthermore, two of the three mediators (satisfaction with getting help and interpersonal relationship) exerted a significant effect on cognitive function (β = 0.177, *p* = 0.008 and β = 0.185, *p* = 0.010 respectively) while the effect of marital satisfaction on cognitive function was insignificant (β = 0.132, *p* = 0.056).

**Table 3:** Multivariate linear regression analysis on the factors associated with cognitive function among the community-dwelling elderly in Jinan, China.

Variables	Model 1		Model 2		Model 3		Model 4		Model 5	
	β	*p*-value	β	*p*-value	β	*p*-value	β	*p*-value	β	*p*-value
Age	−0.275	**<0.001**	−0.265	**<0.001**	−0.264	**<0.001**	−0.262	**<0.001**	−0.263	**<0.001**
Marital status
Currently married	Ref.		Ref.		Ref.		Ref.		Ref.	
Single^a^	−0.062	0.288	−0.062	0.150	−0.048	0.260	−0.051	0.234	−0.043	0.327
Educational level
Illiterate	Ref.				Ref.		Ref.		Ref.	
Primary school	0.271	**<0.001**	0.263	**<0.001**	0.258	**<0.001**	0.263	**<0.001**	0.263	**<0.001**
Middle school	0.460	**<0.001**	0.446	**<0.001**	0.452	**<0.001**	0.452	**<0.001**	0.461	**<0.001**
University or above	0.348	**<0.001**	0.345	**<0.001**	0.350	**<0.001**	0.353	**<0.001**	0.353	**<0.001**
Monthly income^b^
Q1	Ref.		Ref.		Ref.		Ref.		Ref.	
Q2	0.004	0.937	0.010	0.846	0.014	0.786	0.009	0.869	0.014	0.795
Q3	−0.020	0.710	−0.029	0.601	−0.032	0.557	−0.031	0.573	−0.035	0.519
Q4	0.062	0.288	0.056	0.336	0.054	0.351	0.055	0.344	0.046	0.427
Employment status
Currently employed	Ref.		Ref.		Ref.		Ref.		Ref.	
Retired	0.109	0.189	0.114	0.171	0.106	0.199	0.112	0.174	0.108	0.195
Unemployed	0.075	0.391	0.075	0.391	0.083	0.335	0.084	0.331	0.079	0.366
Source of living expenses										
From their own or spouses	Ref.		Ref.		Ref.		Ref.		Ref.	
From their children or other relatives	−0.138	**0.008**	−0.139	**0.007**	−0.140	**0.007**	−0.142	**0.006**	−0.139	**0.007**
From basic living allowances	0.077	0.938	−0.003	0.947	−0.009	0.830	−0.007	0.853	−0.008	0.845
Physical health (PCS)
Poor	Ref.		Ref.		Ref.		Ref.		Ref.	
Good	−0.040	0.346	−0.047	0.268	−0.070	0.102	−0.065	0.130	−0.053	0.208
Inpatient service last year										
No	Ref.		Ref.		Ref.		Ref.		Ref.	
Yes	−0.047	0.257	−0.045	0.279	−0.041	0.322	−0.042	0.304	−0.043	0.303
Self-reported oral health
Low			Ref.		Ref.		Ref.		Ref.	
Moderate			0.072	0.134	0.070	0.144	0.065	0.177	0.071	0.138
High			0.101	**0.040**	0.082	0.096	0.086	0.083	0.094	0.054
Satisfaction with getting help
General or below					Ref.					
Quite satisfied					0.089	0.173				
Very satisfied					0.177	**0.008**				
Satisfaction with interpersonal relationship		
General or below							Ref.			
Quite satisfied							0.142	**0.043**		
Very satisfied							0.185	**0.010**		
Satisfaction with marriage
General or below									Ref.	
Quite satisfied									0.061	0.368
Very satisfied									0.132	0.056
F	13.407	**<0.001**	12.060	**<0.001**	11.341	**<0.001**	11.197	**<0.001**	11.070	**<0.001**
$R_{c}^{2}$	0.254		0.257		0.267		0.264		0.262	
Δ$R_{c}^{2}$	–		0.003		0.010		0.007		0.005	Δ

β: Standardized regression coefficient; PCS: Physical component summary; MCS: Mental component summary.

a Single included those who were unmarried (10, 1.5%), divorced (5, 0.8%), and widowed (58, 8.8%).

b Q1 was the poorest and Q4 was the richest. Bold values indicated statistical significance (*p* ≤ 0.05).

### 3.4. Structural equation modelling analysis

#### 3.4.1. Model fitness indices

**Figure 1:**
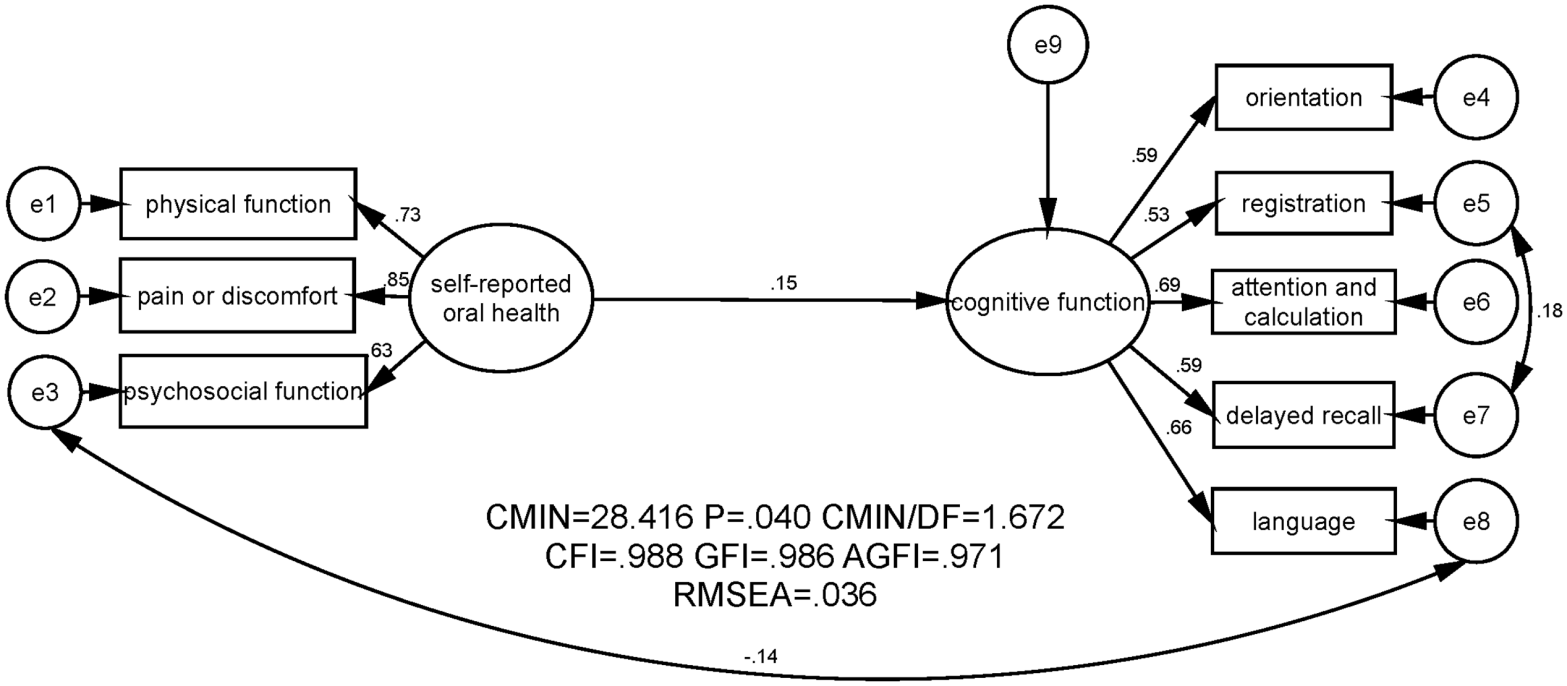
SEM analysis on the association between self-reported oral health and cognitive function among the community-dwelling elderly in Jinan, China. CMIN, Chi-square value; DF, Degree of freedom; CFI, Comparative fit index, GFI, Goodness fit index, AGFI, Adjusted good-ness fit index; RMSEA, Root mean square error of approximation.

**Figure 2:**
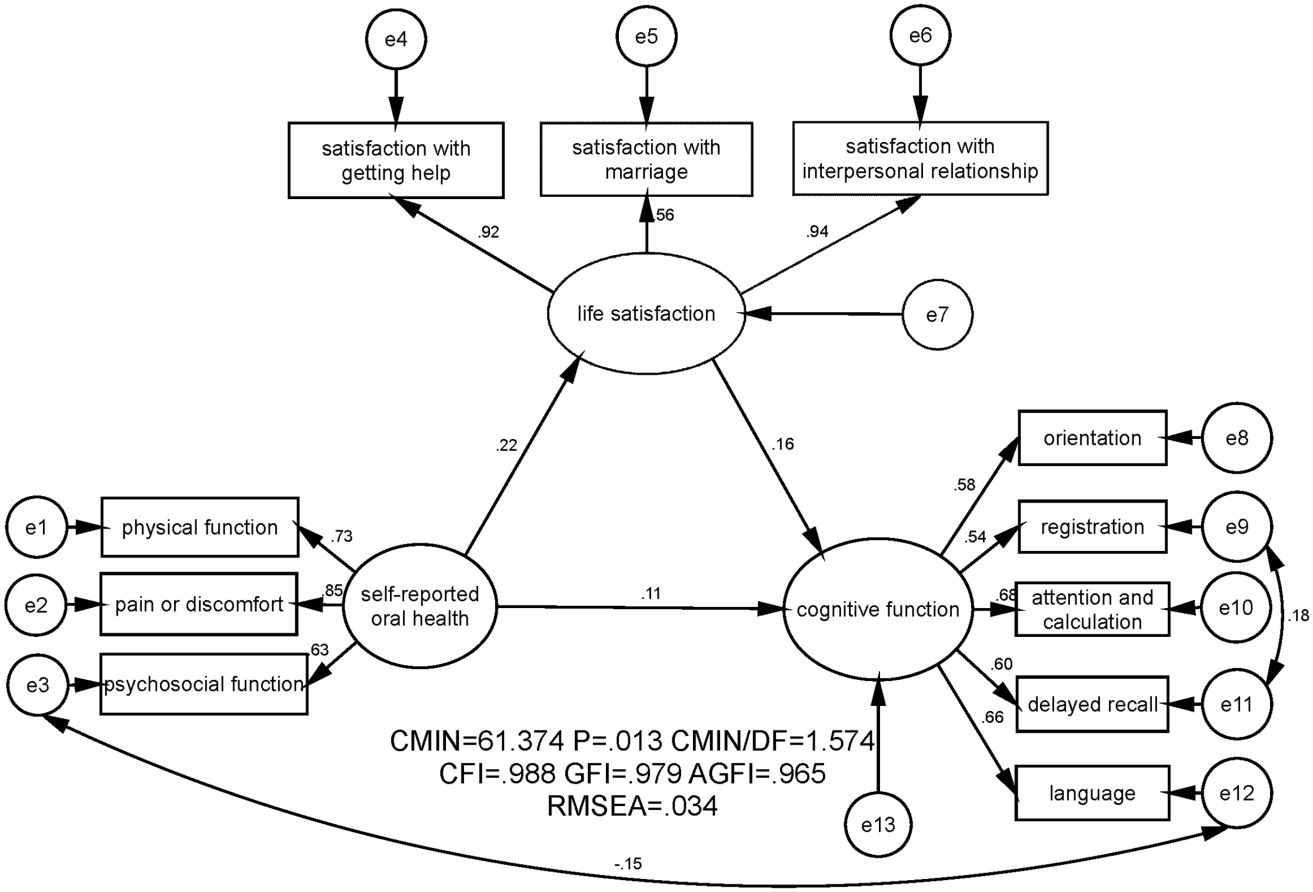
SEM analysis on the mediating effect of life satisfaction on the association between self-reported oral health and cognitive function among the community-dwelling elderly in Jinan, China. CMIN, Chi-square value; DF, Degree of freedom; CFI, Comparative fit index; GFI, Goodness fit index; AGFI, Adjusted good-ness fit index; RMSEA: Root mean square error of approximation.

The two proposed models were shown in Figures 1, 2 respectively. The first model contained two latent variables namely self-reported oral health and cognitive function as well as eight manifest variables. The second model added one more latent variable, namely life satisfaction, and three manifest variables, thus encompassing three latent variables and 11 manifest variables. As shown in Table 4 and Figure 1, in the first model, *χ^2^*/*df* = 1.672, CFI = 0.988, GFI = 0.986, AGFI = 0.971 and RMSEA = 0.036. As shown in Table 4 and Figure 2, in the second model, *χ*^2^/*df* = 1.574, CFI = 0.988, GFI = 0.979, AGFI = 0.965, and RMSEA = 0.034. These results indicated that the two hypothesized models fitted the empirical data obtained in this study well.

**Table 4:** The comparison of the model fit indices for the current model and the cut-off criteria.

Model fit indices	Cut-off criteria	Indices for the first model	Indices for the second model	Decision
$\chi^{2}$	–	28.416*	61.374*	–
$\chi^{2}/df$	<5	1.672	1.574	Good fitting
CFI	>0.90	0.988	0.988	Good fitting
GFI	>0.90	0.986	0.979	Good fitting
AGFI	>0.90	0.971	0.965	Good fitting
RMSEA	<0.08	0.036	0.034	Good fitting

CFI, Comparative fit index; GFI, Goodness fit index; AGFI, Adjusted good-ness fit index; RMSEA, Root mean square error of approximation; *p ≤ 0.05.

#### 3.4.2. Mediating effect of life satisfaction on the relationship between self-reported oral health and cognitive function

Figure 1 illustrated that self-reported oral health had a significant and positive effect on cognitive function (standardized effect was 0.15, *p* ≤ 0.01). Figure 2 showed that when the mediator (life satisfaction) was included, self-reported oral health still exerted an effect on cognitive function; while the coefficient between self-reported oral health and cognitive function decreased (standardized effect was 0.11, *p* ≤ 0.05). Furthermore, with the bias-corrected percentile Bootstrap method, Table 4 presented that the standardized indirect effect of self-reported oral health through life satisfaction was 0.036, and the 95% CI excluded zero (0.010 to 0.075). Thus, the mediating effect of life satisfaction on the association between self-reported oral health and cognitive function was established, and life satisfaction mediated 24% of the total effect of self-reported oral health on cognitive function. Moreover, Figure 2 and Table 5 also displayed a significant and positive association between self-reported oral health and life satisfaction (standardized direct effect was 0.219, 95% CI was 0.122 to 0.319), revealing that older adults in the current study who had better self-reported oral health were more satisfied with their lives. Life satisfaction was significantly and positively related to cognitive function (standardized direct effect was 0.165, 95% CI was 0.040 to 0.287), indicating that the community-dwelling elderly with higher levels of life satisfaction had better cognitive function.

**Table 5:** Standardized coefficients between the self-reported oral health, life satisfaction and cognitive function by using SEM.

Model pathways	Estimated effect	95% CI	
		Lower bounds	Upper bounds
*Total effect*			
Self-reported oral health → Cognitive function	0.150**	0.039	0.274
*Direct effect*			
Self-reported oral health → Cognitive function	0.114*	0.005	0.240
Life satisfaction → Cognitive function	0.165**	0.040	0.287
Self-reported oral health → life satisfaction	0.219***	0.122	0.319
*Indirect effect*			
Self-reported oral health → life satisfaction → Cognitive function	0.036**	0.010	0.075

SEM, Structural equation modelling; CI, Confidence interval; *p ≤ 0.05, **p ≤ 0.01, ***p ≤ 0.001.

## 4. Discussion

This study provided new empirical evidence pertaining to the mediating role of life satisfaction in the association between self-reported oral health and cognitive function. The existence of a positive relationship between self-reported oral health and cognitive function was clearly demonstrated among the sample group of community-dwelling elderly in Jinan, China, with this association shown to be partially mediated by life satisfaction.

The average MMSE score of the older adults in this study was 25.65 ± 4.62, implying that the status of geriatric cognitive function was relatively good. This score was higher than that recently reported in a study conducted in Anhui, China (22.59 ± 6.47) (Xu et al., 2021). A possible explanation was that this survey was conducted in urban areas, while the Anhui based study was mainly completed in rural regions, with the prevalence of cognitive impairment among rural seniors found to be higher than that among urban seniors (Tang et al., 2016). The mean score was also higher than that of a study that used the Chinese Longitudinal Health Longevity Survey database (21.55 ± 2.38) (Zhang Y. T. et al., 2019). One possible reason maybe the difference in participants’ average age (69.66 in our study versus 80.65 in the aforementioned study), since age was an important risk factor for cognitive impairment (Xia et al., 2018). In contrast, the mean MMSE score in this study was lower than that in a previous Singaporean study (27.80 ± 2.80) (Cheong et al., 2021), which may be attributed to the lower mean age (66.6 years old) of the respondents in that study.

Self-reported oral health was found to be significantly and positively correlated with cognitive function, indicating that community-dwelling elderly who reported better self-reported oral health were more likely to be cognitively intact. An English longitudinal study of aging showed that both better oral health and oral health-related quality of life at an earlier phase were predictors of general cognition at a later stage (Kang et al., 2020). Epidemiological evidence also suggested that baseline teeth symptoms caused a decrease in episodic memory and global cognition but not in executive function and working memory among US-based community-dwelling Chinese older individuals (Petrovsky et al., 2019). Several cross-sectional investigations have also provided evidence of the link between self-reported oral health and cognitive function (Jensen et al., 2008).

This study found a positive relationship between life satisfaction and cognitive function, which was consistent with the results of previous studies. A Malaysian cross-sectional study showed that financial satisfaction could increase general life satisfaction and further promote cognitive function among the low-income community-dwelling older adults (Foong et al., 2021). Peitsch et al. conducted a prospective cohort study and demonstrated that lower overall life satisfaction was a strong risk factor for developing dementia within the next five years (Peitsch et al., 2016). One possible interpretation was that people who were less satisfied with their everyday lives were less likely to participate in social activities and build interpersonal networks (Sun et al., 2016), finally resulting in the decrease in cognitive function.

Life satisfaction was found not only directly associated with cognitive function, but also partially mediated the relationship between self-reported oral health and cognitive function. Considering that the indicators of life satisfaction in this study mainly focused on the social dimension, it was possible that the above findings could be interpreted from the perspective of social resources or social networks. According to a previous study, seniors with a fewer number of remained teeth were more likely to report fewer social interactions compared to those whose number of teeth was 20 or above (Igarashi et al., 2020). Thus, poor self-reported oral health status among older adults may influence their interaction with others, predicted social isolation through language function, homeboundness, and appearance (Koyama et al., 2016, 2021) and lowered their satisfaction with interpersonal relationship, which further lead to the decrease of their cognitive function. Additionally, for those families where the elderly with poor self-reported oral health lived, restrictions on the types and amount of suitable food could lead to disagreement on dietary habit and the decrease of the older adults’ marital satisfaction, which furtherly exerted negative effects on cognitive function.

The results of this study also demonstrated that self-reported oral health was positively associated with life satisfaction, which was in line with the findings of a number of previous studies. A German longitudinal study revealed that older adults with better self-reported oral health were more likely to experience higher general life satisfaction and subjective wellbeing (Klotz et al., 2018). The favorable effect of chewing ability on life satisfaction was also observed among middle-aged and older Australian adults (Brennan et al., 2008). However, a study among older people with disabilities found that poor self-reported oral health did not increase the probability of poor life satisfaction (Jensen et al., 2008). This discrepancy might be attributed to a possible adaptive process by those elderly with existing disabilities to the additional oral disease burden.

Finally, this study also found a number of confounding variables that were relevant to cognitive function among the community-dwelling elderly. It was plausible that participants who were older had lower levels of cognitive function, as the results of a previous study indicated that the increasing age were related to higher risk of cognitive impairment (Hussenoeder et al., 2020). Among the participants of this study, those who held a lower educational level also exhibited a lower level of cognitive function; a possible explanation might lie in the fact that a higher level of educational background can contribute to an enhancement in cognitive reserve, further relief from the onset of cognitive decline (Caamaño-Isorna et al., 2006). Respondents who made their livings by their children or relatives had a lower socioeconomic status, thus leading to worse cognitive function.

The results of this study provide the following implications. Firstly, the government should take measures to monitor and improve the self-reported oral health of the elderly at an early stage. It is recommended that self-reported oral health management be introduced as a significant part of the basic public health services. Secondly, communities should pay close attention to the promotion of life satisfaction among seniors. Older people with self-reported oral health problems should be encouraged to communicate with others to improve their level of life satisfaction and cognitive function. Thirdly, family members should attach great importance to the oral symptoms, marital satisfaction and interpersonal relationship of the older individuals, in order to delay the onset of cognitive impairment.

There are also several limitations. Firstly, the questions used to assess life satisfaction predominantly focused on the dimension of social interaction, which could not measure this variable comprehensively. Secondly, due to the practical difficulty of the questionnaire survey, older adults who experienced severe cognitive decline and could not communicate with investigators were excluded from the study, which might have caused selection bias. Thirdly, the scales used in the current study, especially for the GOHAI and MMSE, were self-reported questionnaires, rather than clinical diagnostic standards, which limited the clinical consequences and implications of the results. Fourthly, the study was only conducted in Jinan City, which limited the potential extrapolation of the results. A comparative study of different cities will be conducted in the future. Fifthly, although the correlation between key variables were all statistically significant, some of them were weak. More attention should be paid to this issue in the future. Sixthly, as the current study was conducted during the COVID-19 pandemic, there might be potential effect of the epidemic on oral health and life satisfaction. Lastly, the proportion of participants aged 80 years or above was relatively small, which could influence the extrapolation to the oldest old.

## 5. Conclusion

The level of cognitive function among the community-dwelling elderly in Jinan, China was relatively high. Self-reported oral health was found to be associated with the cognitive function, while life satisfaction exerted a mediating effect on this relationship. Early screening for oral disease and attention to life satisfaction were recommended for policymakers.

## Data availability statement

The raw data supporting the conclusions of this article will be made available by the authors, without undue reservation.

## Ethics statement

The studies involving human participants were reviewed and approved by the Ethical Committee of School of Public Health, Shandong University. The patients/participants provided their written informed consent to participate in this study.

## Author contributions

GL analyzed the data and drafted the manuscript. FK applied for the funding to support this study, designed the study, completed the questionnaire design, supervised and joined the data collection, instructed the writing, statistical analysis, data processing and gave comments on the modification of the manuscript. ZL, YS, JW, XS, DZ, and SL gave many valuable comments on the draft and also polished it. All of the authors read and approved the final manuscript. All authors contributed to the article and approved the submitted version.

## Funding

This study was supported and funded by the National Natural Science Foundation of China (No. 71804094), China Postdoctoral Science Foundation (No. 2016 M592161), Natural Science Foundation of Shandong Province (No. ZR2016GB02), Postdoctoral Science Foundation of Shandong Province (No. 201603021), and Fundamental Research Funds for the Central Universities (No. 2022KJGL01 and No. 2018JC055).

## Conflict of interest

The authors declare that the research was conducted in the absence of any commercial or financial relationships that could be construed as a potential conflict of interest.

## Publisher’s note

All claims expressed in this article are solely those of the authors and do not necessarily represent those of their affiliated organizations, or those of the publisher, the editors and the reviewers. Any product that may be evaluated in this article, or claim that may be made by its manufacturer, is not guaranteed or endorsed by the publisher.
